# The genome sequence of the Northern Grizzled Skipper, *Pyrgus centaureae* (Rambur, 1839) (Lepidoptera: Hesperiidae)

**DOI:** 10.12688/wellcomeopenres.26169.1

**Published:** 2026-03-19

**Authors:** Vlad Dincă, Daniel Linke, Charlotte J. Wright, Joana I. Meier, Mark L. Blaxter, Wellcome Sanger Institute Tree of Life Management, Samples and Laboratory team, Wellcome Sanger Institute Scientific Operations: Sequencing Operations, Wellcome Sanger Institute Tree of Life Core Informatics team, Tree of Life Core Informatics collective, Project Psyche Community

**Affiliations:** 1 Grigore Antipa National Museum of Natural History, Bucharest, Romania; 2 Biology Centre of the Czech Academy of Sciences, Institute of Entomology, České Budějovice, Czech Republic; 3 Tree of Life Programme, Wellcome Sanger Institute, Hinxton, England, UK

**Keywords:** Pyrgus centaureae; Northern Grizzled Skipper; genome sequence; chromosomal; Lepidoptera

## Abstract

We present a genome assembly from a female specimen of *Pyrgus centaureae* (Northern Grizzled Skipper; Arthropoda; Insecta; Lepidoptera; Hesperiidae). The assembly contains two haplotypes with total lengths of 758.98 megabases and 671.41 megabases. Most of haplotype 1 (99.62%) is scaffolded into 31 chromosomal pseudomolecules, including the W and Z sex chromosomes. Haplotype 2 was assembled to scaffold level. The mitochondrial genome has also been assembled, with a length of 15.4 kilobases.

## Species taxonomy

Eukaryota; Opisthokonta; Metazoa; Eumetazoa; Bilateria; Protostomia; Ecdysozoa; Panarthropoda; Arthropoda; Mandibulata; Pancrustacea; Hexapoda; Insecta; Dicondylia; Pterygota; Neoptera; Endopterygota; Amphiesmenoptera; Lepidoptera; Glossata; Neolepidoptera; Heteroneura; Ditrysia; Obtectomera; Hesperioidea; Hesperiidae; Pyrginae; *Pyrgus*; *Pyrgus centaureae* (Rambur, 1839) (NCBI:txid1666702).

## Background

*P. centaureae*, commonly known as the Northern Grizzled Skipper, is a small butterfly species in the family Hesperiidae with a Holarctic distribution. In Europe and Asia, it is present from northern Fennoscandia through northern Russia, the Urals, and the Altai Mountains. In the Nearctic, its range includes Canada and Alaska, extending south to New Mexico in the Rocky Mountains and North Carolina in the Appalachian Mountains ( de Jong, 1975; Lotts & Naberhaus, 2025; Tolman & Lewington, 2009). It inhabits marshes, tundra, open hillsides, or shrublands at elevations from 0 to 2 600 m, while being mostly absent from coastal areas.

Adults of *P. centaureae* have a wingspan of 25–33 mm, with veins lined in white on the ventral side, which is unusual for *Pyrgus* species. Additionally, they display a distinctive white spot near the costa on the dorsal hindwing ( Tolman & Lewington, 2009). Larval development takes two years in the subarctic, with eggs deposited during June to July (in southern North American populations, adults fly considerably earlier, from March to June ( Chazal *et al.*, 2004)). In the subarctic, larvae overwinter as second-instar caterpillars. In the second year, larvae complete development and overwinter as a pupa. Notably, larval host plants switch during development, at least in European subspecies: first-instar larvae prefer dwarf birch leaves ( *Betula nana*) and shift to cloudberry leaves ( *Rubus chamaemorus*) after the first moult ( Wickman, 2012). Larvae construct shelters from folded birch or cloudberry leaves ( Wickman, 2012). In addition to cloudberry, larvae may feed on other plants in the Rose family, such as wild strawberries ( *Fragaria virginiana*) and cinquefoils ( *Potentilla canadensis* or *P. diversifolia*) ( Lotts & Naberhaus, 2025).

Genetically, *P. centaureae* is split between Palaearctic and Nearctic lineages. A recently described subspecies, *P. centaureae dzekh* Gorbunov, 2007, which occurs in eastern Siberia, has also been recorded from Alaska ( Zhang *et al.*, 2021), suggesting multiple relatively recent trans-Beringian dispersal events.

The IUCN assesses *P. centaureae* as Vulnerable, with decreasing populations ( van Swaay *et al.*, 2025a; van Swaay *et al.*, 2025b). In North America, some subspecies require conservation action due to fragmented ranges, such as *P. c. wyandot* inhabiting the Appalachian Mountains ( Chazal *et al.*, 2004). Outside Europe, populations are generally secure, although the species may be rare in parts of its range, especially at the periphery ( Lotts & Naberhaus, 2025).

**Figure 1.**
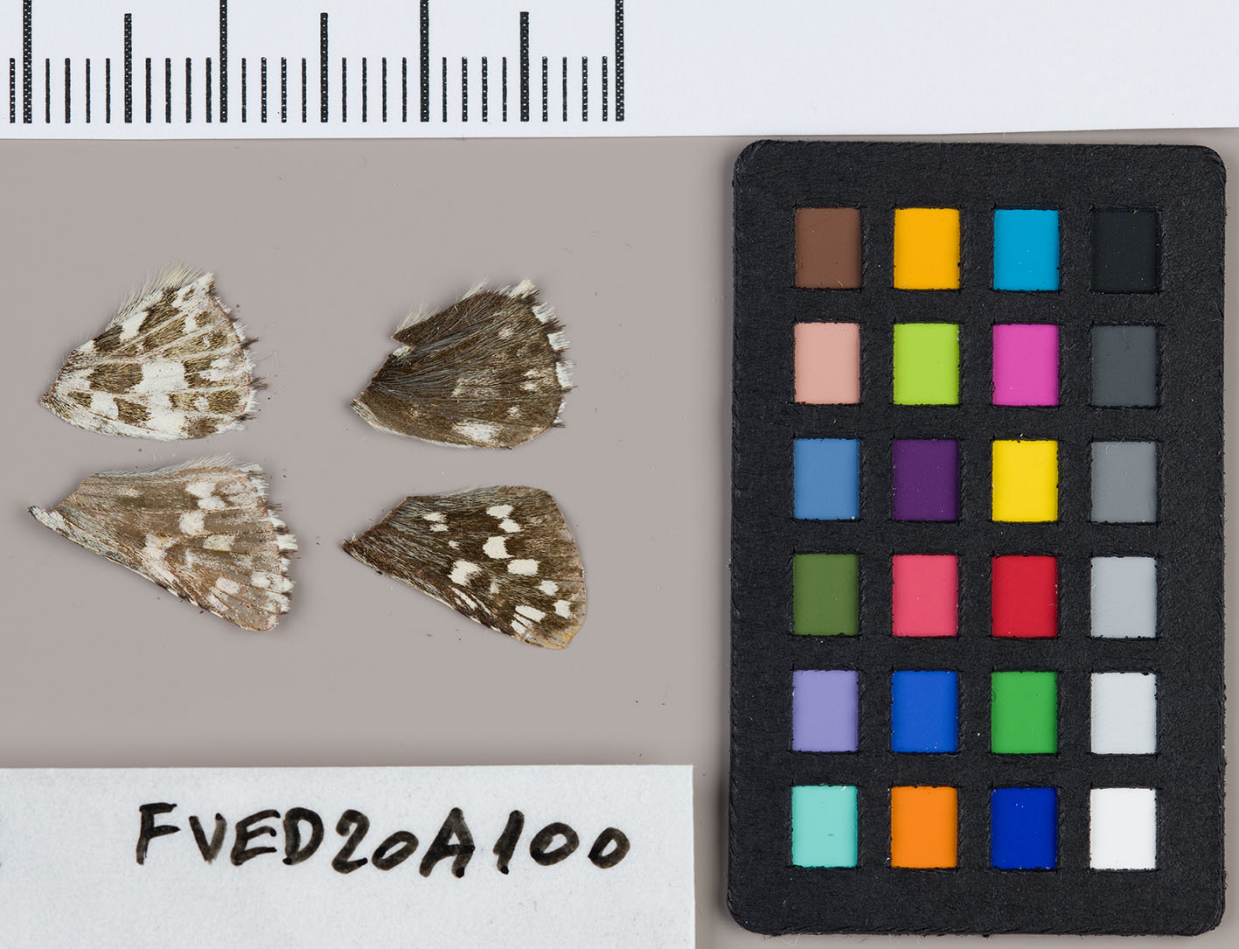
Voucher photograph of the *Pyrgus centaureae* (ilPyrCent2) specimen used for genome sequencing.

We present a chromosome-level genome sequence of *P. centaureae*, sequenced as part of Project Psyche ( Wright *et al.*, 2025). The sequence data were derived from a female specimen ( Figure 1) collected from Rauhala, Lapland, Finland.

## Methods

### Sample acquisition

The specimen used for genome sequencing was an adult female *P. centaureae* (specimen ID SAN28000256, ToLID ilPyrCent2; Figure 1), collected from Rauhala, Lapland, Finland (latitude 67.8829, longitude 24.2031; elevation 265 m) on 2020-06-22. The specimen was collected and identified by Vlad Dincă.

### Nucleic acid extraction

Protocols for high molecular weight (HMW) DNA extraction developed at the Wellcome Sanger Institute (WSI) Tree of Life Core Laboratory are available on protocols.io ( Howard *et al.*, 2025). The ilPyrCent2 sample was weighed and triaged to determine the appropriate extraction protocol. Tissue from the whole organism was homogenised by powermashing using a PowerMasher II tissue disruptor.

HMW DNA was extracted in the WSI Scientific Operations core using the Automated MagAttract v2 protocol. DNA was sheared into an average fragment size of 12–20 kb following the Megaruptor®3 for LI PacBio protocol. Sheared DNA was purified by automated SPRI (solid-phase reversible immobilisation). The concentration of the sheared and purified DNA was assessed using a Nanodrop spectrophotometer and Qubit Fluorometer using the Qubit dsDNA High Sensitivity Assay kit. Fragment size distribution was evaluated by running the sample on the FemtoPulse system. For this sample, the final post-shearing DNA had a Qubit concentration of 41.23 ng/μL and a yield of 1 937.81 ng, with a fragment size of 16.2 kb.

RNA was extracted from whole organism tissue of ilPyrCent2 in the Tree of Life Laboratory at the WSI using the RNA Extraction: Automated MagMax™ *mir*Vana protocol. The RNA concentration was assessed using a Nanodrop spectrophotometer and a Qubit Fluorometer using the Qubit RNA Broad-Range Assay kit. Analysis of the integrity of the RNA was done using the Agilent RNA 6000 Pico Kit and Eukaryotic Total RNA assay.

### PacBio HiFi library preparation and sequencing

Library preparation and sequencing were performed at the WSI Scientific Operations core. Libraries were prepared using the SMRTbell Prep Kit 3.0 (Pacific Biosciences, California, USA), according to the manufacturer’s instructions. The kit includes reagents for end repair/A-tailing, adapter ligation, post-ligation SMRTbell bead clean-up, and nuclease treatment. Size selection and clean-up were performed using diluted AMPure PB beads (Pacific Biosciences). DNA concentration was quantified using a Qubit Fluorometer v4.0 (ThermoFisher Scientific) and the Qubit 1X dsDNA HS assay kit. Final library fragment size was assessed with the Agilent Femto Pulse Automated Pulsed Field CE Instrument (Agilent Technologies) using the gDNA 55 kb BAC analysis kit.

The sample was sequenced on a Revio instrument (Pacific Biosciences). The prepared library was normalised to 2 nM, and 15 μL was used for making complexes. Primers were annealed and polymerases bound to generate circularised complexes, following the manufacturer’s instructions. Complexes were purified using 1.2X SMRTbell beads, then diluted to the Revio loading concentration (200–300 pM) and spiked with a Revio sequencing internal control. The sample was sequenced on a Revio 25 M SMRT cell. The SMRT Link software (Pacific Biosciences), a web-based workflow manager, was used to configure and monitor the run and to carry out primary and secondary data analysis. Specimen details, sequencing platforms, and data yields are summarised in Table 1.

**Table 1.** Specimen and sequencing data for BioProject PRJEB80080.

Platform	PacBio HiFi	Hi-C	RNA-seq
**ToLID**	ilPyrCent2	ilPyrCent2	ilPyrCent2
**Specimen ID**	SAN28000256	SAN28000256	SAN28000256
**BioSample (source individual)**	SAMEA115575572	SAMEA115575572	SAMEA115575572
**BioSample (tissue)**	SAMEA115575600	SAMEA115575600	SAMEA115575600
**Tissue**	whole organism	whole organism	whole organism
**Instrument**	Revio	Illumina NovaSeq X	Illumina NovaSeq X
**Run accessions**	ERR13660092	ERR13670051	ERR14986714
**Read count total**	2.22 million	677.22 million	132.97 million
**Base count total**	23.75 Gb	102.26 Gb	20.08 Gb

### Hi-C

***Sample preparation and crosslinking***

The Hi-C sample was prepared from 20–50 mg of frozen tissue from the whole organism of the ilPyrCent2 sample using the Arima-HiC v2 kit (Arima Genomics). Following the manufacturer’s instructions, tissue was fixed and DNA crosslinked using TC buffer to a final formaldehyde concentration of 2%. The tissue was homogenised using the Diagnocine Power Masher-II. Crosslinked DNA was digested with a restriction enzyme master mix, biotinylated, and ligated. Clean-up was performed with SPRISelect beads before library preparation. DNA concentration was measured with the Qubit Fluorometer (Thermo Fisher Scientific) and Qubit HS Assay Kit. The biotinylation percentage was estimated using the Arima-HiC v2 QC beads.

***Hi-C library preparation and sequencing***

Biotinylated DNA constructs were fragmented using a Covaris E220 sonicator and size selected to 400–600 bp using SPRISelect beads. DNA was enriched with Arima-HiC v2 kit Enrichment beads. End repair, A-tailing, and adapter ligation were carried out with the NEBNext Ultra II DNA Library Prep Kit (New England Biolabs), following a modified protocol where library preparation occurs while DNA remains bound to the Enrichment beads. Library amplification was performed using KAPA HiFi HotStart mix and a custom Unique Dual Index (UDI) barcode set (Integrated DNA Technologies). Depending on sample concentration and biotinylation percentage determined at the crosslinking stage, libraries were amplified with 10–16 PCR cycles. Post-PCR clean-up was performed with SPRISelect beads. Libraries were quantified using the AccuClear Ultra High Sensitivity dsDNA Standards Assay Kit (Biotium) and a FLUOstar Omega plate reader (BMG Labtech).

Prior to sequencing, libraries were normalised to 10 ng/μL. Normalised libraries were quantified again to create equimolar and/or weighted 2.8 nM pools. Pool concentrations were checked using the Agilent 4200 TapeStation (Agilent) with High Sensitivity D500 reagents before sequencing. Sequencing was performed using paired-end 150 bp reads on the Illumina NovaSeq X.

Specimen details, sequencing platforms, and data yields are summarised in Table 1.

### RNA-seq library preparation and sequencing

Libraries were prepared using the NEBNext ^®^ Ultra™ II Directional RNA Library Prep Kit for Illumina (New England Biolabs), following the manufacturer’s instructions. Poly(A) mRNA in the total RNA solution was isolated using oligo (dT) beads, converted to cDNA, and uniquely indexed; 14 PCR cycles were performed. Libraries were size-selected to produce fragment lengths of 100–300 bp. Libraries were quantified, normalised, pooled to a final concentration of 2.8 nM, and diluted to 150 pM for loading. Sequencing was carried out on the Illumina NovaSeq X to generate 150-bp paired-end reads.

### Genome assembly

Prior to assembly of the PacBio HiFi reads, a database of *k*-mer counts ( *k* = 31) was generated from the filtered reads using FastK. GenomeScope2 ( Ranallo-Benavidez *et al.*, 2020) was used to analyse the *k*-mer frequency distributions, providing estimates of genome size, heterozygosity, and repeat content.

The HiFi reads were assembled using Hifiasm in Hi-C phasing mode ( Cheng *et al.*, 2021, 2022), producing two haplotypes. Hi-C reads ( Rao *et al.*, 2014) were mapped to the primary contigs using bwa-mem2 ( Vasimuddin *et al.*, 2019). Contigs were further scaffolded with Hi-C data in YaHS ( Zhou *et al.*, 2023), using the --break option for handling potential misassemblies. The scaffolded assemblies were evaluated using Gfastats ( Formenti *et al.*, 2022), BUSCO ( Manni *et al.*, 2021) and MERQURY.FK ( Rhie *et al.*, 2020).

The mitochondrial genome was assembled using MitoHiFi ( Uliano-Silva *et al.*, 2023), which runs MitoFinder ( Allio *et al.*, 2020) and uses these annotations to select the final mitochondrial contig and to ensure the general quality of the sequence.

### Assembly curation

The assembly was decontaminated using the Assembly Screen for Cobionts and Contaminants ( ASCC) pipeline. TreeVal was used to generate the flat files and maps for use in curation. Manual curation was conducted primarily in PretextView and HiGlass ( Kerpedjiev *et al.*, 2018). Scaffolds were visually inspected and corrected as described by Howe *et al*. (2021). Manual corrections included 12 breaks and 30 joins. This reduced the scaffold count by 11.7%, reduced scaffold N50 by 0.6%, and increased the total assembly length by 1.1%. The curation process is described at https://gitlab.com/wtsi-grit/rapid-curation . PretextSnapshot was used to generate a Hi-C contact map of the final assembly.

### Assembly quality assessment

The Merqury.FK tool ( Rhie *et al.*, 2020), run in a Singularity container ( Kurtzer *et al.*, 2017), was used to evaluate *k*-mer completeness and assembly quality for both haplotypes using the *k*-mer database ( *k* = 31) computed prior to genome assembly. The analysis outputs included assembly QV scores and completeness statistics.

The genome was analysed using the BlobToolKit pipeline, a Nextflow ( Di Tommaso *et al.*, 2017) implementation of the earlier Snakemake version ( Challis *et al.*, 2020). The pipeline aligns PacBio reads using minimap2 ( Li, 2018) and SAMtools ( Danecek *et al.*, 2021) to generate coverage tracks. It runs BUSCO ( Manni *et al.*, 2021) using lineages identified from the NCBI Taxonomy ( Schoch *et al.*, 2020). For the three domain-level lineages, BUSCO genes are aligned to the UniProt Reference Proteomes database ( Bateman *et al.*, 2023) using DIAMOND blastp ( Buchfink *et al.*, 2021). The genome is divided into chunks based on the density of BUSCO genes from the closest taxonomic lineage, and each chunk is aligned to the UniProt Reference Proteomes database with DIAMOND blastx. Sequences without hits are chunked using seqtk and aligned to the NT database with blastn ( Altschul *et al.*, 1990). The BlobToolKit suite consolidates all outputs into a blobdir for visualisation. The BlobToolKit pipeline was developed using nf-core tooling ( Ewels *et al.*, 2020) and MultiQC ( Ewels *et al.*, 2016), with containerisation through Docker ( Merkel, 2014) and Singularity ( Kurtzer *et al.*, 2017).

We used lep_busco_painter to paint Merian elements along chromosomes ( Wright *et al.*, 2024). Merian elements represent the 32 ancestral linkage groups in Lepidoptera. The painting process utilised BUSCO gene locations from the lepidoptera_odb10 set ( Kriventseva *et al.*, 2019) and chromosome lengths from NCBI Datasets. Each complete BUSCO gene (both single-copy and duplicated) was assigned to a Merian element based on a reference database, then plotted along chromosomes drawn to scale.

## Genome sequence report

### Sequence data

**Figure 2.**
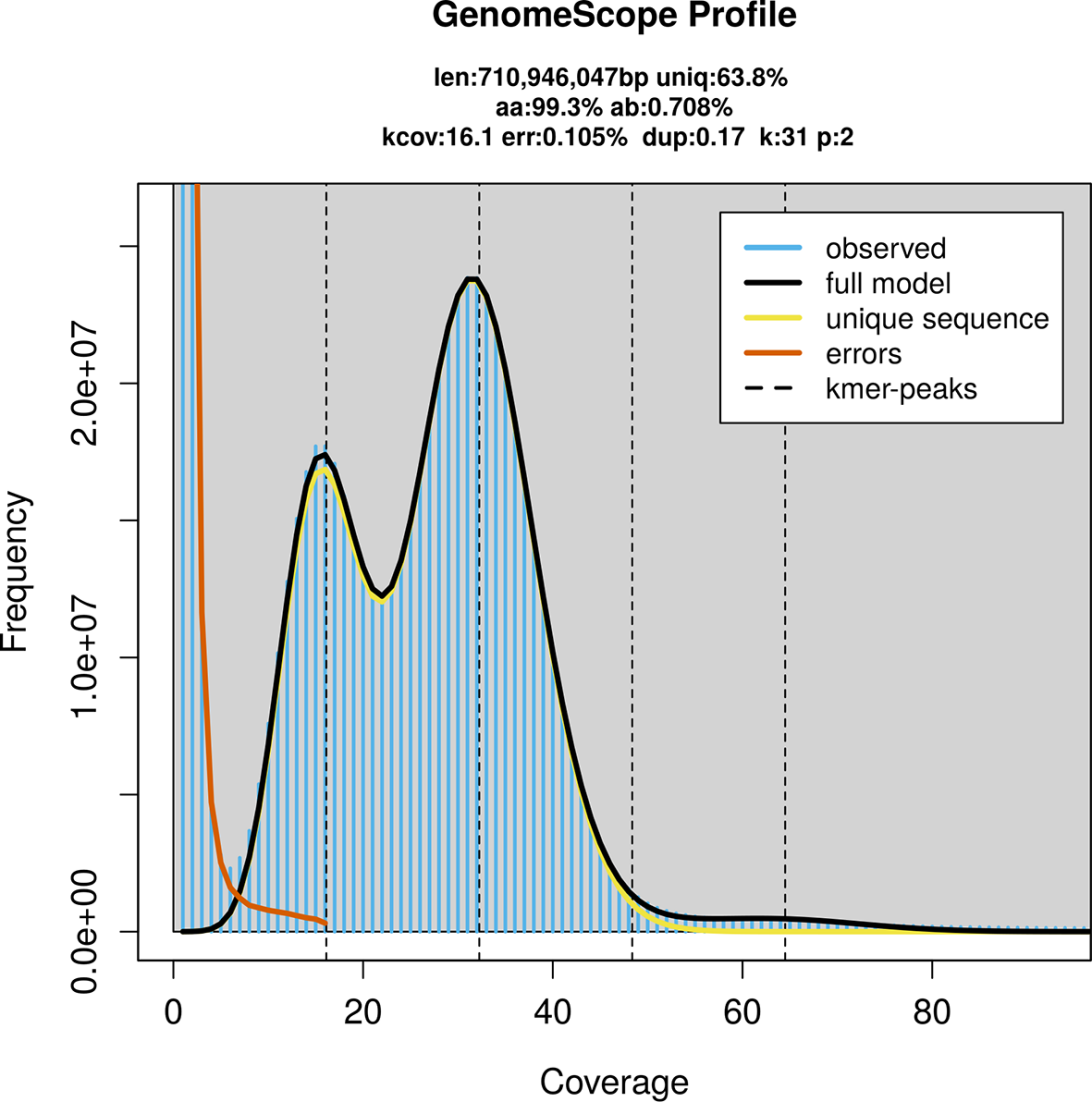
Frequency distribution of *k*-mers generated using GenomeScope2. The plot shows observed and modelled *k*-mer spectra, providing estimates of genome size, heterozygosity, and repeat content based on unassembled sequencing reads.

PacBio sequencing of the *P. centaureae* specimen generated 23.75 Gb (gigabases) from 2.22 million reads, which were used to assemble the genome. GenomeScope2.0 analysis estimated the haploid genome size at 711.77 Mb, with a heterozygosity of 0.71% and repeat content of 36.20% ( Figure 2). These estimates guided expectations for the assembly. Based on the estimated genome size, the sequencing data provided approximately 32× coverage. Hi-C sequencing produced 102.26 Gb from 677.22 million reads, which were used to scaffold the assembly. RNA sequencing data were also generated and are available in public sequence repositories. Table 1 summarises the specimen and sequencing details.

### Assembly statistics

The genome was assembled into two haplotypes using Hi-C phasing. Haplotype 1 was curated to chromosome level, while haplotype 2 was assembled to scaffold level. The final assembly has a total length of 758.98 Mb in 127 scaffolds, with 274 gaps, and a scaffold N50 of 26.32 Mb ( Table 2).

**Table 2.** Genome assembly statistics.

**Assembly name**	ilPyrCent2.hap1.1	ilPyrCent2.hap2.1
**Assembly accession**	GCA_964275275.1	GCA_964275205.1
**Assembly level**	chromosome	scaffold
**Span (Mb)**	758.98	671.41
**Number of chromosomes**	31	scaffold-level
**Number of contigs**	401	328
**Contig N50**	4.49 Mb	4.54 Mb
**Number of scaffolds**	127	89
**Scaffold N50**	26.32 Mb	25.96 Mb
**Longest scaffold length (Mb)**	55.72	-
**Sex chromosomes**	W and Z	-
**Organelles**	Mitochondrion: 15.4 kb	-

**Figure 3.**
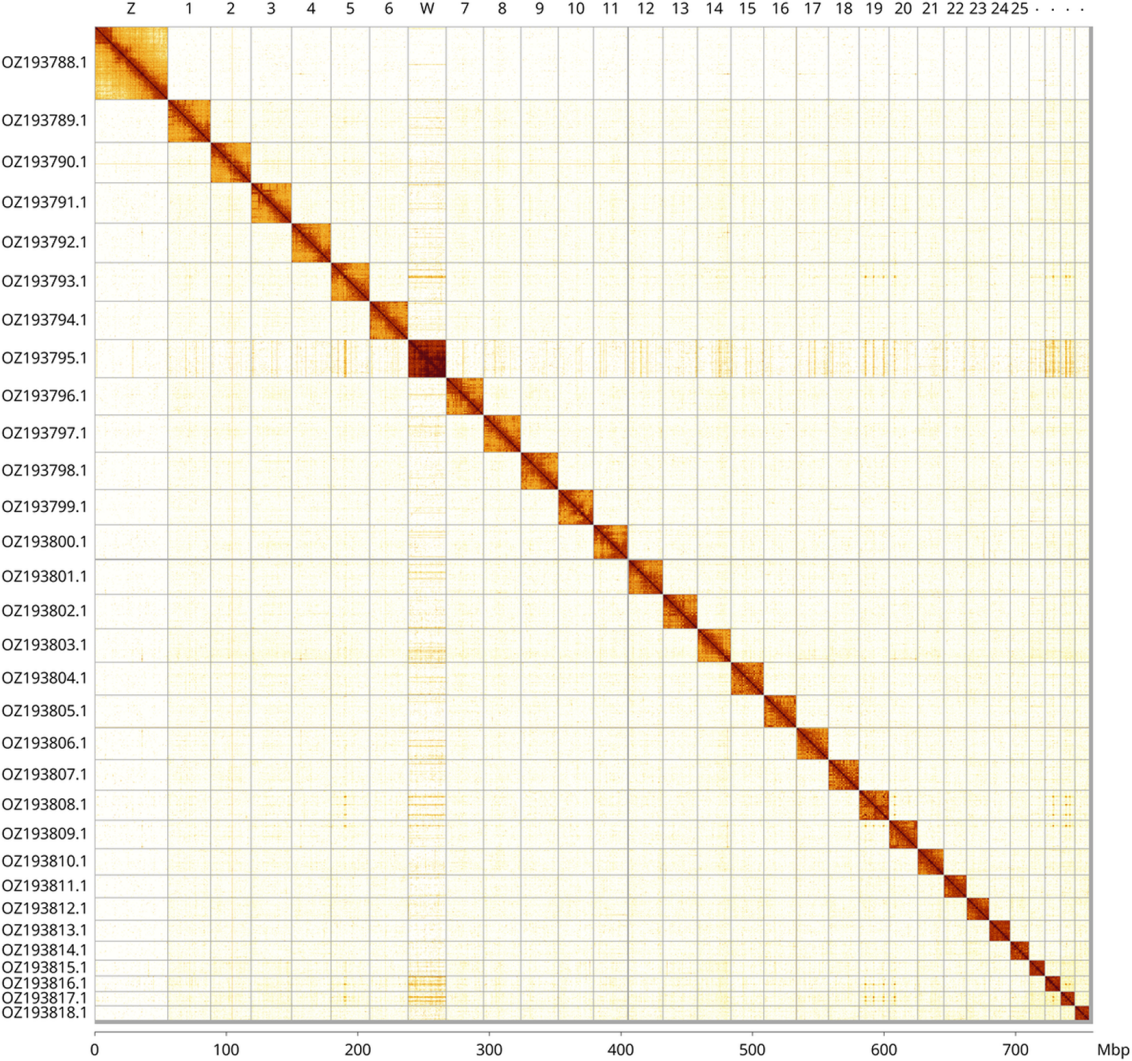
Hi-C contact map of the *Pyrgus centaureae* genome assembly. Assembled chromosomes are shown in order of size and labelled along the axes, with a megabase scale shown below. The plot was generated using PretextSnapshot.

**Figure 4.**
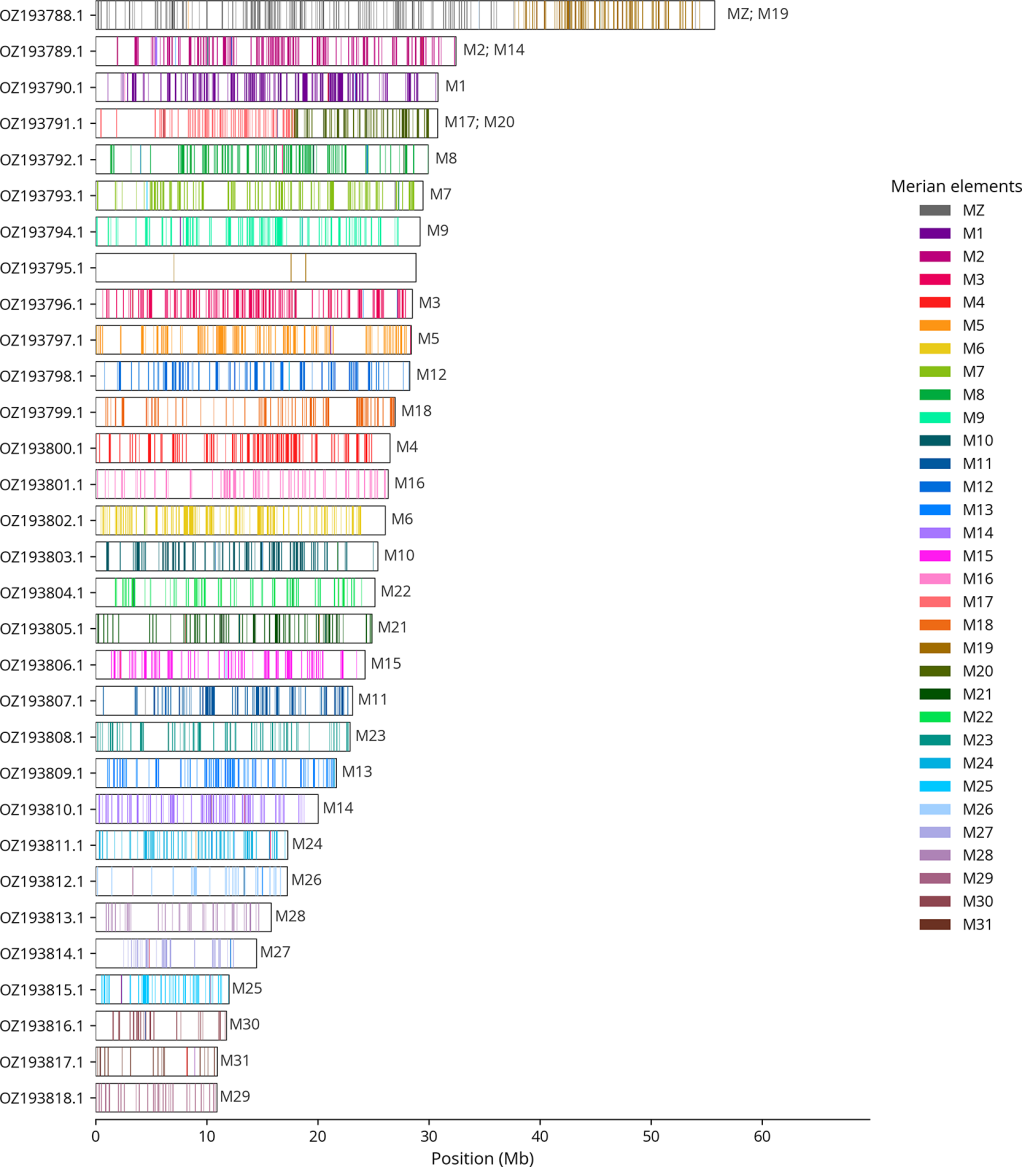
Merian elements painted across chromosomes in the ilPyrCent2.hap1.1 assembly of *Pyrgus centaureae.* Chromosomes are drawn to scale, with the positions of orthologues shown as coloured bars. Each orthologue is coloured by the Merian element that it belongs to. All orthologues which could be assigned to Merian elements are shown.

Most of the assembly sequence (99.62%) was assigned to 31 chromosomal-level scaffolds, representing 29 autosomes and the W and Z sex chromosomes. These chromosome-level scaffolds, confirmed by Hi-C data, are named according to size ( Figure 3 and Table 3). Chromosome painting with Merian elements illustrates the distribution of orthologues along chromosomes and highlights patterns of chromosomal evolution relative to Lepidopteran ancestral linkage groups ( Figure 4).

**Table 3.** Chromosomal pseudomolecules in the haplotype 1 genome assembly of *Pyrgus centaureae* ilPyrCent2.

INSDC accession	Molecule	Length (Mb)	GC%	Assigned Merian elements
OZ193789.1	1	32.42	36.50	M14;M2
OZ193790.1	2	30.82	37	M1
OZ193791.1	3	30.77	37	M17;M20
OZ193792.1	4	29.94	36.50	M8
OZ193793.1	5	29.45	36.50	M7
OZ193794.1	6	29.21	36.50	M9
OZ193796.1	7	28.51	36.50	M3
OZ193797.1	8	28.41	36.50	M5
OZ193798.1	9	28.27	36.50	M12
OZ193799.1	10	26.95	36.50	M18
OZ193800.1	11	26.49	37	M4
OZ193801.1	12	26.32	36.50	M16
OZ193802.1	13	26.06	36.50	M6
OZ193803.1	14	25.41	37	M10
OZ193804.1	15	25.14	36.50	M22
OZ193805.1	16	24.88	36.50	M21
OZ193806.1	17	24.26	37	M15
OZ193807.1	18	23.12	37	M11
OZ193808.1	19	22.90	37	M23
OZ193809.1	20	21.69	37	M13
OZ193810.1	21	20.02	37	M14
OZ193811.1	22	17.30	37.50	M24
OZ193812.1	23	17.24	37	M26
OZ193813.1	24	15.80	37.50	M28
OZ193814.1	25	14.50	37.50	M27
OZ193815.1	26	11.99	38	M25
OZ193816.1	27	11.78	39.50	M30
OZ193817.1	28	10.94	38	M31
OZ193818.1	29	10.92	38	M29
OZ193795.1	W	28.85	37.50	-
OZ193788.1	Z	55.72	36.50	M19;MZ

The mitochondrial genome was also assembled (length 15.4 kb, OZ193819.1). This sequence is included as a contig in the multifasta file of the genome submission and as a standalone record.

### Assembly quality metrics

**Figure 5.**
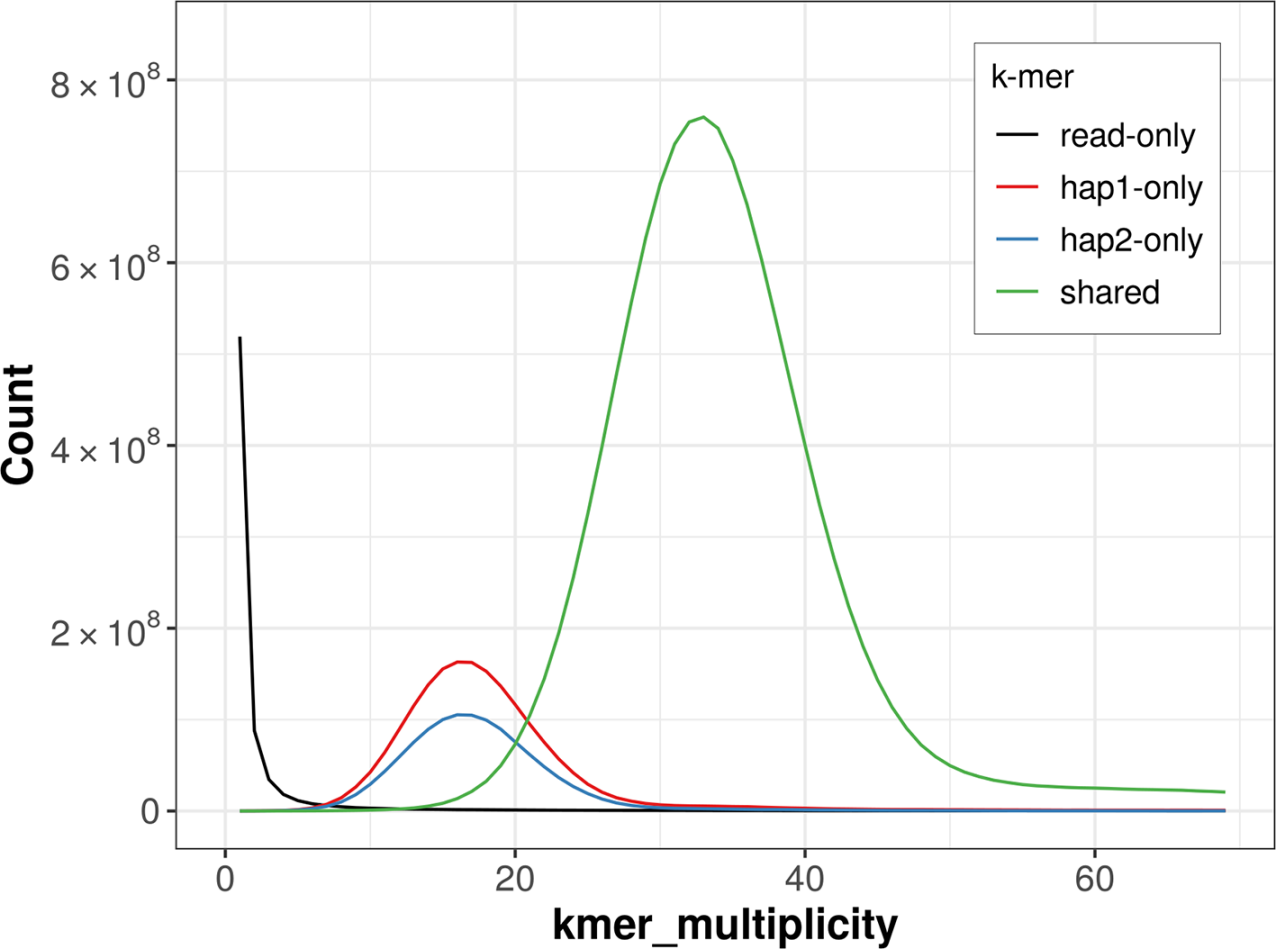
Evaluation of *k*-mer completeness using MerquryFK. This plot illustrates the recovery of *k*-mers from the original read data in the final assemblies. The horizontal axis represents *k*-mer multiplicity, and the vertical axis shows the number of *k*-mers. The black curve represents *k*-mers that appear in the reads but are not assembled. The green curve (the homozygous peak) corresponds to *k*-mers shared by both haplotypes and the red and blue curves (the heterozygous peaks) show *k*-mers found only in one of the haplotypes.

For haplotype 1, the estimated QV is 62.4, and for haplotype 2, 62.0. When the two haplotypes are combined, the assembly achieves an estimated QV of 62.2. The *k*-mer completeness is 86.99% for haplotype 1, 80.25% for haplotype 2, and 99.31% for the combined haplotypes ( Figure 5).

BUSCO analysis using the lepidoptera_odb10 reference set ( *n* = 5 286) identified 98.5% of the expected gene set (single = 97.8%, duplicated = 0.6%) in haplotype 1. For haplotype 2, BUSCO analysis identified 91.8% of the expected gene set (single = 91.5%, duplicated = 0.3%).

**Figure 6.**
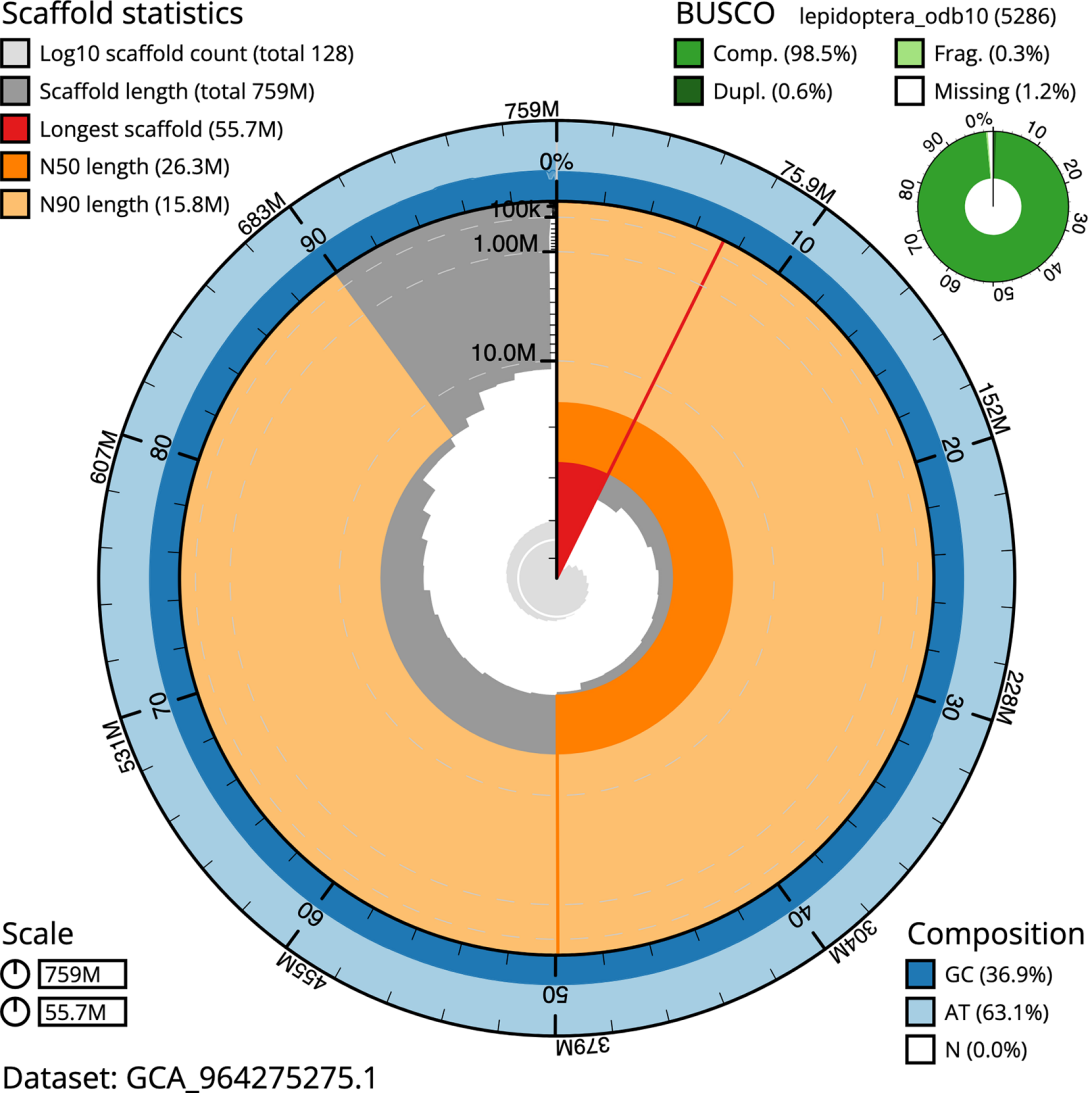
Assembly metrics for ilPyrCent2.hap1.1. The BlobToolKit snail plot provides an overview of assembly metrics and BUSCO gene completeness. The circumference represents the length of the whole genome sequence, and the main plot is divided into 1,000 bins around the circumference. The outermost blue tracks display the distribution of GC, AT, and N percentages across the bins. Scaffolds are arranged clockwise from longest to shortest and are depicted in dark grey. The longest scaffold is indicated by the red arc, and the deeper orange and pale orange arcs represent the N50 and N90 lengths. A light grey spiral at the centre shows the cumulative scaffold count on a logarithmic scale. A summary of complete, fragmented, duplicated, and missing BUSCO genes in the set is presented at the top right. An interactive version of this figure can be accessed on the BlobToolKit viewer.

**Figure 7.**
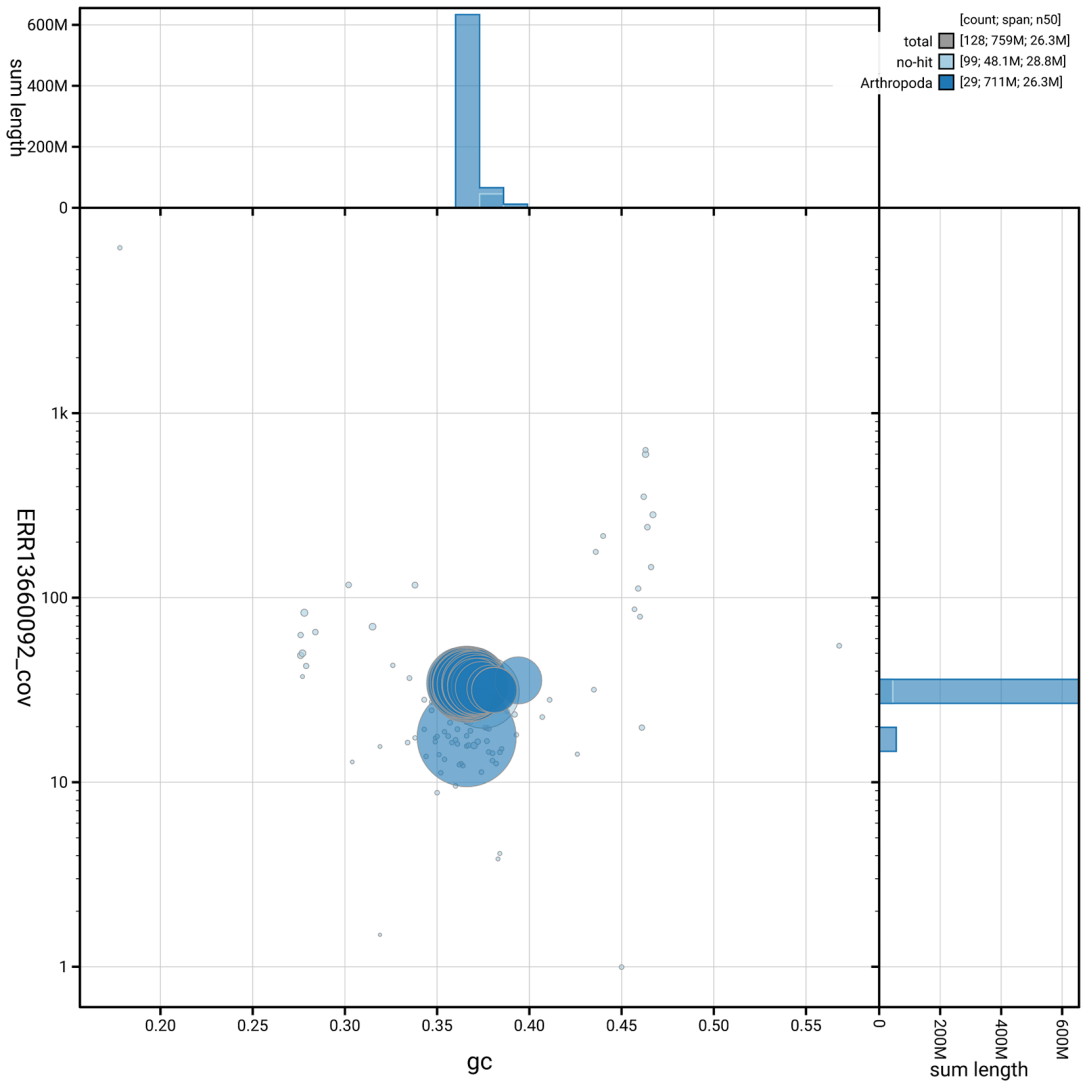
BlobToolKit GC-coverage plot for ilPyrCent2.hap1.1. Blob plot showing sequence coverage (vertical axis) and GC content (horizontal axis). The circles represent scaffolds, with the size proportional to scaffold length and the colour representing phylum membership. The histograms along the axes display the total length of sequences distributed across different levels of coverage and GC content. An interactive version of this figure is available on the BlobToolKit viewer.

The snail plot in Figure 6 summarises the scaffold length distribution and other assembly statistics for haplotype 1. The blob plot in Figure 7 shows the distribution of scaffolds by GC proportion and coverage for haplotype 1.

Table 4 lists the assembly metric benchmarks adapted from Rhie *et al.* (2021) and the Earth BioGenome Project Report on Assembly Standards January 2026. The EBP metric, calculated for the haplotype 1, is **6.C.Q62**, meeting the recommended reference standard.

**Table 4.** Earth Biogenome Project summary metrics for the *Pyrgus centaureae* assembly.

Measure	Value	Benchmark
EBP summary (haplotype 1)	6.C.Q62	6.C.Q40
Contig N50 length	4.49 Mb	≥ 1 Mb
Scaffold N50 length	26.32 Mb	= chromosome N50
Consensus quality (QV)	Haplotype 1: 62.4; haplotype 2: 62.0; combined: 62.2	≥ 40
*k*-mer completeness	Haplotype 1: 86.99%; Haplotype 2: 80.25%; combined: 99.31%	≥ 95%
BUSCO	C:98.5% [S:97.8%; D:0.6%]; F:0.3%; M:1.2%; n:5 286	S > 90%; D < 5%
Percentage of assembly assigned to chromosomes	99.62%	≥ 90%

**Notes:** EBP summary uses log10(Contig N50); chromosome-level (C) or log10(Scaffold N50); Q (Merqury QV). BUSCO: C = complete; S = single-copy; D = duplicated; F = fragmented; M = missing; n = orthologues.

## Author information

Contributors are listed at the following links:

• Members of the Wellcome Sanger Institute Tree of Life Management, Samples and Laboratory team
• Members of Wellcome Sanger Institute Scientific Operations – Sequencing Operations
• Members of the Wellcome Sanger Institute Tree of Life Core Informatics team
• Members of the Tree of Life Core Informatics collective
• Members of the Project Psyche Community

## Wellcome Sanger Institute – Legal and Governance

The materials that have contributed to this genome note have been supplied by a Tree of Life collaborator. The Wellcome Sanger Institute employs a process whereby due diligence is carried out proportionate to the nature of the materials themselves, and the circumstances under which they have been/are to be collected and provided for use. The purpose of this is to address and mitigate any potential legal and/or ethical implications of receipt and use of the materials as part of the research project, and to ensure that in doing so, we align with best practice wherever possible. The overarching areas of consideration are:

• Ethical review of provenance and sourcing of the material.
• Legality of collection, transfer and use (national and international).

Each transfer of samples is undertaken according to a Research Collaboration Agreement or Material Transfer Agreement entered into by the Tree of Life collaborator, Genome Research Limited (operating as the Wellcome Sanger Institute), and in some circumstances, other Tree of Life collaborators.

## Data Availability

European Nucleotide Archive: *P. centaureae.* Accession number PRJEB80080. The genome sequence is released openly for reuse. The *P. centaureae* genome sequencing initiative is part of the Sanger Institute Tree of Life Programme (PRJEB43745) and Project Psyche (PRJEB71705). All raw sequence data and the assembly have been deposited in INSDC databases. The genome will be annotated using available RNA-Seq data and presented through Ensembl at the European Bioinformatics Institute. Raw data and assembly accession identifiers are reported in Tables 1 and 2. Pipelines used for genome assembly at the WSI Tree of Life are available at https://pipelines.tol.sanger.ac.uk/pipelines. Table 5 lists software versions used in this study.

## References

[ref1] Allio R Schomaker-Bastos A Romiguier J : MitoFinder: Efficient automated large-scale extraction of mitogenomic data in target enrichment phylogenomics. *Mol. Ecol. Resour.* 2020;20(4):892–905. 10.1111/1755-0998.13160 32243090 PMC7497042

[ref2] Altschul SF Gish W Miller W : Basic Local Alignment Search Tool. *J. Mol. Biol.* 1990;215(3):403–410. 10.1016/S0022-2836(05)80360-2 2231712

[ref3] Bateman A Martin M-J Orchard S : UniProt: The Universal Protein Knowledgebase in 2023. *Nucleic Acids Res.* 2023;51(D1):D523–D531. 10.1093/nar/gkac1052 36408920 PMC9825514

[ref4] Buchfink B Reuter K Drost H-G : Sensitive protein alignments at tree-of-life scale using DIAMOND. *Nat. Methods.* 2021;18(4):366–368. 10.1038/s41592-021-01101-x 33828273 PMC8026399

[ref5] Challis R Richards E Rajan J : BlobToolKit – interactive quality assessment of genome assemblies. *G3 Genes|Genomes|Genetics.* 2020;10(4):1361–1374. 10.1534/g3.119.400908 32071071 PMC7144090

[ref6] Chazal AC Roble SM Hobson CS : Status of the Appalachian grizzled skipper ( *Pyrgus centaureae wyandot*) in Virginia. *Banisteria: a journal devoted to the natural history of Virginia.* 2004;24:15–22.

[ref7] Cheng H Concepcion GT Feng X : Haplotype-resolved *de novo* assembly using phased assembly graphs with Hifiasm. *Nat. Methods.* 2021;18(2):170–175. 10.1038/s41592-020-01056-5 33526886 PMC7961889

[ref8] Cheng H Jarvis ED Fedrigo O : Haplotype-resolved assembly of diploid genomes without parental data. *Nat. Biotechnol.* 2022;40(9):1332–1335. 10.1038/s41587-022-01261-x 35332338 PMC9464699

[ref9] Danecek P Bonfield JK Liddle J : Twelve years of SAMtools and BCFtools. *GigaScience.* 2021;10(2). 10.1093/gigascience/giab008 33590861 PMC7931819

[ref10] Jong R de : Notes on the genus *Pyrgus* (Lepidoptera, Hesperiidae). 1975;1–11.

[ref11] Di Tommaso P Chatzou M Floden EW : Nextflow enables reproducible computational workflows. *Nat. Biotechnol.* 2017;35(4):316–319. 10.1038/nbt.3820 28398311

[ref12] Ewels P Magnusson M Lundin S : MultiQC: Summarize analysis results for multiple tools and samples in a single report. *Bioinformatics.* 2016;32(19):3047–3048. 10.1093/bioinformatics/btw354 27312411 PMC5039924

[ref13] Ewels PA Peltzer A Fillinger S : The nf-core framework for community-curated bioinformatics pipelines. *Nat. Biotechnol.* 2020;38(3):276–278. 10.1038/s41587-020-0439-x 32055031

[ref14] Formenti G Abueg L Brajuka A : Gfastats: Conversion, evaluation and manipulation of genome sequences using assembly graphs. *Bioinformatics.* 2022;38(17):4214–4216. 10.1093/bioinformatics/btac460 35799367 PMC9438950

[ref15] Howard C Denton A Jackson B : On the path to reference genomes for all biodiversity: Lessons learned and laboratory protocols created in the Sanger Tree of Life core laboratory over the first 2000 species. *bioRxiv.* 2025. 10.1101/2025.04.11.648334 PMC12548527 41129326

[ref16] Howe K Chow W Collins J : Significantly improving the quality of genome assemblies through curation. *GigaScience.* 2021;10(1). 10.1093/gigascience/giaa153 33420778 PMC7794651

[ref17] Kerpedjiev P Abdennur N Lekschas F : HiGlass: Web-based visual exploration and analysis of genome interaction maps. *Genome Biol.* 2018;19(1):125. 10.1186/s13059-018-1486-1 30143029 PMC6109259

[ref18] Kriventseva EV Kuznetsov D Tegenfeldt F : OrthoDB v10: Sampling the diversity of animal, plant, fungal, protist, bacterial and viral genomes for evolutionary and functional annotations of orthologs. *Nucleic Acids Res.* 2019;47(D1):D807–D811. 10.1093/nar/gky1053 30395283 PMC6323947

[ref19] Kurtzer GM Sochat V Bauer MW : Singularity: Scientific containers for mobility of compute. *PLoS One.* 2017;12(5):e0177459. 10.1371/journal.pone.0177459 28494014 PMC5426675

[ref20] Li H : Minimap2: Pairwise alignment for nucleotide sequences. *Bioinformatics.* 2018;34(18):3094–3100. 10.1093/bioinformatics/bty191 29750242 PMC6137996

[ref21] Lotts K Naberhaus T : Grizzled skipper, Pyrgus centaureae (Rambur, [1842]). 2025. Butterflies; Moths of North America. Reference Source

[ref22] Manni M Berkeley MR Seppey M : BUSCO update: Novel and streamlined workflows along with broader and deeper phylogenetic coverage for scoring of eukaryotic, prokaryotic, and viral genomes. *Mol. Biol. Evol.* 2021;38(10):4647–4654. 10.1093/molbev/msab199 34320186 PMC8476166

[ref23] Merkel D : Docker: Lightweight Linux containers for consistent development and deployment. *Linux J.* 2014;2014(239). 10.5555/2600239.2600241

[ref24] Ranallo-Benavidez TR Jaron KS Schatz MC : GenomeScope 2.0 and Smudgeplot for reference-free profiling of polyploid genomes. *Nat. Commun.* 2020;11(1):1432. 10.1038/s41467-020-14998-3 32188846 PMC7080791

[ref25] Rao SSP Huntley MH Durand NC : A 3D map of the human genome at kilobase resolution reveals principles of chromatin looping. *Cell.* 2014;159(7):1665–1680. 10.1016/j.cell.2014.11.021 25497547 PMC5635824

[ref26] Rhie A McCarthy SA Fedrigo O : Towards complete and error-free genome assemblies of all vertebrate species. *Nature.* 2021;592(7856):737–746. 10.1038/s41586-021-03451-0 33911273 PMC8081667

[ref27] Rhie A Walenz BP Koren S : Merqury: Reference-free quality, completeness, and phasing assessment for genome assemblies. *Genome Biol.* 2020;21(1). 10.1186/s13059-020-02134-9 32928274 PMC7488777

[ref28] Schoch CL Ciufo S Domrachev M : NCBI taxonomy: A comprehensive update on curation, resources and tools. *Database.* 2020;2020:baaa062. 10.1093/database/baaa062 32761142 PMC7408187

[ref29] Tolman T Lewington R : *Collins Butterfly Guide: The Most Complete Guide to the Butterflies of Britain and Europe.* HarperCollins Publishers;2009.

[ref30] Uliano-Silva M Ferreira JGRN Krasheninnikova K : MitoHiFi: A Python pipeline for mitochondrial genome assembly from PacBio high fidelity reads. *BMC Bioinformatics.* 2023;24(1):288. 10.1186/s12859-023-05385-y 37464285 PMC10354987

[ref31] Swaay C van Ellis S Warren M : Pyrgus carlinae. 2025a. 10.2305/IUCN.UK.2025-1.RLTS.T173307A211447452.en

[ref32] Swaay C van Warren M Ellis S : European Red List of butterflies: Measuring the pulse of European biodiversity. 2025b. 10.2779/935927

[ref33] Vasimuddin M Misra S Li H : Efficient architecture-aware acceleration of BWA-MEM for multicore systems. *2019 IEEE International Parallel and Distributed Processing Symposium (IPDPS).* IEEE;2019;314–24. 10.1109/IPDPS.2019.00041

[ref34] Wickman P-O : Värdväxtbyte hos myrvisslaren, *Pyrgus centaureae* (Lepidoptera: Hesperiidae). *Entomol Tidskr.* 2012;133(3):93–100.

[ref35] Wright CJ Stevens L Mackintosh A : Comparative genomics reveals the dynamics of chromosome evolution in Lepidoptera. *Nat. Ecol. Evol.* 2024;8(4):777–790. 10.1038/s41559-024-02329-4 38383850 PMC11009112

[ref36] Wright CJ Wahlberg N Vila R : Project Psyche: Reference genomes for all Lepidoptera in Europe. *Trends Ecol. Evol.* 2025;40(12):1234–1250. 10.1016/j.tree.2025.10.007 41309387

[ref37] Zhang J Cong Q Shen J : Genomics-guided refinement of butterfly taxonomy. *Taxon. Rep. Int. Lepidoptera Surv.* 2021;9(3):1–55. 10.5281/zenodo.5630311 Reference Source 35098146 PMC8794009

[ref38] Zhou C McCarthy SA Durbin R : YaHS: Yet another Hi-C scaffolding tool. *Bioinformatics.* 2023;39(1). 10.1093/bioinformatics/btac808 PMC9848053 36525368

