# Supplementary material for: The genome sequence of the Northern Grizzled Skipper, Pyrgus centaureae (Rambur, 1839) (Lepidoptera: Hesperiidae)
Source: Wellcome Open Res. 2026 Mar 19;11:184. [Version 1] doi: 10.12688/wellcomeopenres.26169.1 (PMC13109703; doi:10.12688/wellcomeopenres.26169.1)
Supplement: Peer review history

# Reviewer report: Reviewer response for version 1

**DOI:** 10.21956/wellcomeopenres.28821.r152560

**Published:** 2026-06-01

**Authors:** Gulab D Khedkar

**Affiliations:** 1 Dr Babasaheb Ambedkar Marathwada University, Aurangabad, Maharashtra, India

**Summary of evaluation**

 The manuscript presents a high-quality genome assembly and comprehensive genomic resource for *Pyrgus centaureae *with strong technical rigor and well-documented analytical workflows. The study is timely and valuable, particularly considering the ecological and conservation relevance of this species. The assembly quality, inclusion of haplotype-resolved genomes, and integration of mitochondrial genome data substantially enhance the utility of the dataset for future evolutionary, ecological, and comparative genomic investigations in Lepidoptera. The manuscript is clearly written, technically sound, and supported by robust methodologies and appropriate bioinformatic pipelines.

 The rationale for generating the genomic resource is adequately described, and the datasets are presented in a transparent and accessible manner. The methodological details are sufficient to ensure reproducibility. The use of PacBio HiFi sequencing, Hi-C scaffolding, and multiple quality assessment tools has resulted in a highly reliable genome assembly. In particular, the chromosomal-level scaffolding and incorporation of sex chromosomes significantly strengthen the overall quality and long-term utility of the resource.

 A few minor suggestions could further improve the manuscript. First, the authors may consider expanding the discussion on how this genome resource could specifically contribute to future research in Lepidoptera systematics, adaptation, phylogeography, conservation genomics, and population-level analyses. A brief comparison with currently available skipper butterfly genomes may also help contextualize the significance of this assembly within Hesperiidae genomics.

 Additionally, a concise note on possible applications of the dataset for climate resilience, habitat fragmentation studies, or comparative chromosome evolution would further broaden the impact of the work.

 Overall, this is a valuable and well-executed genomic resource paper that will serve as an important reference for future biodiversity genomics studies. The manuscript meets the standards for indexing, and I recommend its acceptance after minor revision.

Are sufficient details of methods and materials provided to allow replication by others?

Yes

Is the rationale for creating the dataset(s) clearly described?

Yes

Are the datasets clearly presented in a useable and accessible format?

Yes

Are the protocols appropriate and is the work technically sound?

Yes

Reviewer Expertise:

Genomics, conservation and polulation genetics

I confirm that I have read this submission and believe that I have an appropriate level of expertise to confirm that it is of an acceptable scientific standard.

# Reviewer report: Reviewer response for version 1

**DOI:** 10.21956/wellcomeopenres.28821.r152567

**Published:** 2026-04-24

**Authors:** Pritha Dey

**Affiliations:** 1 National Centre for Biological Sciences-TIFR, Bengaluru, India

This manuscript presents a genome assembly for the Northern Grizzled Skipper, including both haplotypes and the mitochondrial genome. The study provides a valuable genomic resource for this species, particularly with the high level of completeness in haplotype 1, where the majority of the assembly is scaffolded into chromosomal pseudomolecules, including both sex chromosomes.

 The assembly appears to be of good quality, and the inclusion of both haplotypes is a notable strength. The authors also added the IUCN assessment of the species which adds value to this work.

 However,  how this resource advances research in Lepidoptera or Hesperiidae is not clearly highlighted. Expanding on potential applications of the genome (e.g., evolutionary, ecological, or conservation studies) would also strengthen the impact of the work.

Are sufficient details of methods and materials provided to allow replication by others?

Yes

Is the rationale for creating the dataset(s) clearly described?

Yes

Are the datasets clearly presented in a useable and accessible format?

Yes

Are the protocols appropriate and is the work technically sound?

Yes

Reviewer Expertise:

Evolutionary ecology

I confirm that I have read this submission and believe that I have an appropriate level of expertise to confirm that it is of an acceptable scientific standard.

# Reviewer report: Reviewer response for version 1

**DOI:** 10.21956/wellcomeopenres.28821.r152569

**Published:** 2026-04-23

**Authors:** Melody Clark

**Affiliations:** 1 Natural Environment Research Council, Cambridge, UK

This Data Note describes the sequencing and assembly of a female specimen of the Northern Grizzled Skipper, Pyrgus centaureae. This species has a Holartic distribution with the biogeography and feeding preferences of larvae well described. The North American population is labelled as "Vulnerable" due to habitat fragmentation, which provides added urgency for the need to sequence the genome.  A haplotype level assembly is described with haplotype 1 scaffolded onto 31 pseudochromosomes including the W and Z sex chromosomes; haplotype 2 is to scaffold level. The mitochondrial genome has also been assembled. Thus, the genome is of very high quality. All the methods of DNA and RNA extraction are described and rely on well-known commercial kits, the methodologies are described and follow standard Sanger pipelines which are all in the public domain. PacBio and Hi-C were used to produce the final genome and the bioinformatics pipelines are again well described and follow standard Sanger practice, as do the descriptive analyses of the haplotypes. Application of Merian elements further annotates the genome relative to the ancestral Lepidopteran lineage.

Are sufficient details of methods and materials provided to allow replication by others?

Yes

Is the rationale for creating the dataset(s) clearly described?

Yes

Are the datasets clearly presented in a useable and accessible format?

Yes

Are the protocols appropriate and is the work technically sound?

Yes

Reviewer Expertise:

transcriptomics and genomics of non-model species

I confirm that I have read this submission and believe that I have an appropriate level of expertise to confirm that it is of an acceptable scientific standard.
